# Neutrophil Heterogeneity in Cancer: From Biology to Therapies

**DOI:** 10.3389/fimmu.2019.02155

**Published:** 2019-09-20

**Authors:** Pacôme Lecot, Matthieu Sarabi, Manuela Pereira Abrantes, Julie Mussard, Leo Koenderman, Christophe Caux, Nathalie Bendriss-Vermare, Marie-Cécile Michallet

**Affiliations:** ^1^Department of Immunity, Virus, and Inflammation (IVI), Centre de Recherche en Cancérologie de Lyon, Centre Léon Bérard, University of Lyon, Université Claude Bernard Lyon 1, INSERM 1052, CNRS 5286, Lyon, France; ^2^Department of Respiratory Medicine and Center of Translational Immunology, University Medical Center Utrecht, Utrecht, Netherlands

**Keywords:** neutrophil (PMN), cancer, immunotherapy, MDSC (myeloid-derived suppressor cells), G-MDSC (granulocytic MDSC), tumor-associated neutrophils (TANs), subsets

## Abstract

Neutrophils have been extensively described in the pathophysiology of autoimmune and infectious diseases. Accumulating evidence also suggests the important role of neutrophils in cancer progression through their interaction with cancer and immune cells in blood and in the tumor microenvironment (TME). Most studies have described neutrophils as key drivers of cancer progression, due to their involvement in various tumor promoting functions including proliferation, aggressiveness, and dissemination, as well as in immune suppression. However, such studies were focusing on late-stages of tumorigenesis, in which chronic inflammation had already developed. The role of tumor-associated neutrophils (TANs) at early stages of tumor development remains poorly described, though recent findings indicate that early-stage TANs may display anti-tumor properties. Beyond their role at tumor site, evidence supported by NLR retrospective studies and functional analyses suggest that blood neutrophils could also actively contribute to tumorigenesis. Hence, it appears that the phenotype and functions of neutrophils vary greatly during tumor progression, highlighting their heterogeneity. The origin of pro- or anti-tumor neutrophils is generally believed to arise following a change in cell state, from resting to activated. Moreover, the fate of neutrophils may also involve distinct differentiation programs yielding various subsets of pro or anti-tumor neutrophils. In this review, we will discuss the current knowledge on neutrophils heterogeneity across different tissues and their impact on tumorigenesis, as well as neutrophil-based therapeutic strategies that have shown promising results in pre-clinical studies, paving the way for the design of neutrophil-based next generation immunotherapy.

## Circulating Neutrophils in Cancer

Aside from the molecular signals driving cancer, several studies have demonstrated the contribution of the host-driven inflammatory response to tumor progression and/or to treatment outcome (1–4). Neutrophils are key players in the inflammatory response. They are released into the bloodstream after maturation and differentiation from the bone marrow reservoir (5). The production of neutrophils has been estimated to range from 1 to 2 × 10^11^ cells per day at steady state in a healthy adult. Neutrophils represent 50–70% of all circulating leucocytes in humans, while they account for 10–25% in mice (6).

### Neutrophil Count to Lymphocyte Count Ratio (NLR)

Circulating neutrophil counts are systematically monitored by oncologists during cancer management, owing to chemotherapy-induced neutropenia, which makes patients more vulnerable to life-threatening infections (7, 8). Availability of blood cell counts from retrospective analyses has led to numerous reviews and meta-analyses investigating the prognostic value of the neutrophil count (or preferably neutrophil-to-lymphocyte ratio, also called NLR) in both localized or metastatic contexts (9–12). Hence, over the past decade, literature on NLR has grown steadily. For instance, it is now well-acknowledged that NLR elevation is strongly associated with poor median progression-free survival (mPFS) and median overall survival (mOS) regardless of tumor type, stage of the disease or treatment (9). Interestingly, among metastatic patients, NLR has a prognostic value before chemotherapy and after subsequent lines of treatment in more advanced disease management (11). Optimal NLR cut-off values used to determine increased risk of mortality vary greatly (between 1.9 and 9.21) across studies (12). If we consider NLR as a continuous variable, each of its incremental increases in standard deviation is associated with a 35% increase in the risk of mortality. Moreover, patient follow-up duration influences NLR, as illustrated by the fact that the largest differences observed across patient prognostic groups occur within the first 12 months of follow-up (11). Beside confirming the baseline prognostic value of NLR, its early decrease following only one cycle of chemotherapy appears to be of good prognosis in multiple pathologies: (i) colorectal cancer (13, 14); (ii) mesothelioma; (iii) triple-negative breast cancer (15); (iv) docetaxel-treated patients bearing lung, prostate, head and neck or breast cancers (16); (v) advanced pancreatic adenocarcinoma; and (vi) in peripheral T-cell lymphomas (17).

Conversely, other retrospective studies did not confirm the predictive value of NLR across different randomized chemotherapy arms in colorectal cancer (18), or in advanced biliary tract carcinomas, in which low NLR values during chemotherapy were not associated with significant improvement in survival (only high NLR baseline values that decreased under chemotherapy predicted a significant better mOS) (19). NLR pretreatment values were not predictive of outcome in prostate cancer patients treated with docetaxel (20).

Variation in NLR values during targeted therapy seems to be an interesting biomarker of response in metastatic renal cell carcinoma patients (19). Templeton et al. reported a retrospective analysis of 1,199 patients treated with targeted therapies (bevacizumab, axitinib, sorafenib, sunitinib, temsirolimus) from the metastatic renal cell carcinoma database consortium and highlighted the predictive value of NLR variation between baseline (before targeted therapy commenced) and at 6 weeks (±2 weeks). They used a validation cohort of 4,350 patients from a prospective clinical trial. Compared to no change, a decrease in NLR exceeding 25% was associated with a significant improvement in the response rate, mPFS and mOS, while an increase was predictive of poor outcome. Of note, the highest response rates were observed in groups with low baseline NLR values that remained low at 6 weeks, though a good response rate was also reported in groups with an NLR superior to 3 which declined below 2.25 after the administration of targeted therapy (21). Similar observations were described in non-small cell lung carcinomas treated with gefitinib or erlotinib (22–24), advanced gastro-intestinal stromal tumors (25), advanced soft-tissue sarcoma treated with pazopanib (26), and hepatocellular carcinomas treated with sorafenib (27, 28).

The emergence of anti-CTLA4 and anti-PD(L)1 immunotherapies provides new hope in cancer management. However, due to an overall response rate below 40% (29, 30) and to treatment costs, stratification of patients to identify the best candidate for immunotherapy has become a challenge. Readily available total blood count has enabled the evaluation of NLR in patients receiving immunotherapy, such as in advanced melanoma in which NLR in pretreated patients was identified as an independent marker of response (31–33), even when NLR was recorded during treatment (34). Similarly, in metastatic non-small cell lung carcinomas under anti-PD-1 therapy (30, 35), higher baseline NLR values were associated with a lower response rate (36). In patients with various advanced solid tumors candidates to phase I trials combining PD-1/PD-L1 inhibitors, a low NLR was correlated with response to treatment and improved OS, but not with increased immune toxicity (37).

Cancer-associated systemic inflammation often characterized by a high NLR, associated with a poor prognosis, was thought to occur only at late stages of tumorigenesis (38, 39). Evidence also suggests that NLR may increase at early-stage (stage I and stage II, separately) before treatment, and retains its poor prognostic significance in various cancer types including colon cancer (40), tongue cancer (41), breast cancer (42), and liver cancer (43). Early systemic modifications may therefore occur at early-stages of tumorigenesis.

Discrepancies across studies may be due to the level of heterogeneity of the populations studied (e.g., primary tumor, stage of disease, patient features: medical history or concomitant medication), limitations of retrospective reports, wide variation in NLR cut-off values, as well as in the dynamic assessment of NLR during treatment, and/or a lack of specificity of NLR (neutrophil count gathering immature neutrophils that might be released in the context of inflammation and expected circulating mature neutrophils). Interestingly, studies assessing the prognostic and/or predictive values of NLR in a wide variety of conditions (disease, stages, histology, treatment…) argue in favor of its utility. This correlation of NLR with clinical outcome suggests that changes in NLR may be linked with broader modifications beyond the tumor microenvironment. Hence, beyond being a relevant clinical biomarker, increase in NLR with disease progression highlights the importance of considering the systemic environment and not simply the tumor for a deeper understanding of biological mechanisms underlying cancer progression. It is possible that the tumor itself secretes factors into the bloodstream, which thereby act on bone marrow to skew hematopoiesis toward granulocytic lineages. Tumor may also release danger/damaged-associated molecules (DAMP) that might be a target for infiltrating neutrophils. Hence, the means by which an increase in NLR promotes cancer progression remains to be elucidated.

### T-Cell Suppressive Circulating Neutrophils

Beyond their increase in the peripheral blood of cancer patients, which was associated with a poor prognosis, subsets of circulating neutrophils were reported to display tumor-promoting functions, inferring a causative role in cancer progression rather than just a consequence of the disease. The most-extensively described tumor-promoting function of circulating neutrophils remains their ability to suppress T-cell proliferation and/or activation *in vitro*. These T-cell suppressive neutrophils are classically termed granulocytic myeloid-derived suppressor cells (G-MDSCs) for both humans and mice. G-MDSCs were documented to expand in tumor-bearing hosts compared to healthy subjects (44, 45). In cancer patients, G-MDSCs are CD11b^+^ CD14^−^ CD66b^+^ CD15^hi^ expressing-cells that are enriched in the low-density neutrophils (LDNs) fraction present within the peripheral blood mononuclear cell (PBMCs) ring, unlike normal density neutrophils (NDNs), which are found in the granulocyte pellet of ficolled blood (46–49). In mice, G-MDSC correspond to CD11b^+^ Ly6C^int^ Ly6G^hi^-expressing cells present within the spleen or the tumor (45, 50, 51), and only few studies refer mouse G-MDSCs to LDNs (44). Although LDNs are the best described neutrophil subset(s) in the blood of cancer patients, there is currently no clear LDN-specific biomarker(s). The scavenger receptor Lox1 was recently reported to be expressed by a subset of LDNs (48). Moreover, those LDNs contained both mature and immature neutrophils (44, 49). These findings suggest that LDNs remain a heterogeneous population of circulating neutrophils that need to be further characterized.

Recent studies conducted in human subjects, revealed new subsets of T-cell suppressive circulating neutrophils based on their stage of maturity. Activated mature neutrophils defined as CD11c^bright^ CD62L^dim^ CD11b^bright^ CD16^bright^ cells in healthy volunteers challenged with LPS systemically (52) and CD66b^+^ CD11b^bright^ CD16^bright^ mature LDNs in cancer patients (49) were reported to suppress T-cell proliferation. Both studies show that mature neutrophils inhibit interferon gamma production (IFNγ) by activated T-cells (49, 52). High level of CD66b^+^ CD11b^bright^ CD16^bright^ mature LDNs strongly correlated with adverse outcome in head and neck cancer (49). Interestingly, Evrard et al. demonstrated in a murine model of pancreatic cancer that the concentration of blood Ly6G^high^ CD101^−^ immature neutrophils was significantly greater in mice with a high tumor burden compared to the low tumor burden group, whereas this was not the case for mature neutrophils (53). In the same line, another group showed that the adoptive transfer of unipotent, committed human CD66b^+^ CD117^+^ neutrophil progenitor (hNeP) in immune deficient NSG-M3 mice accelerated osteosarcoma tumor growth compared with the transfer of committed monocyte progenitors (54). Surprisingly in this study, *in vitro* co-culture of either hNeP or mature bone marrow neutrophils with T-cells, activated the latter based on the upregulation of CD69, rather than inhibiting T-cell activation compared to control (54). The precise mechanisms by which immature circulating neutrophils contribute to tumor growth remain unknown.

The suppression of T-cell proliferation by circulating neutrophils has been attributed to the release of different molecules. Reactive oxygen species (ROS) and Arginase 1 are the two most extensively described neutrophil-derived T-cell suppressive factors (46–48, 52, 55). In humans, both factors require a CD18/Mac-1 immunological synapse between neutrophils and T-cells to display suppressive functions (52, 55). Circulating neutrophils appear to suppress T-cell proliferation via reversible cell cycle arrest rather than induction of apoptosis, as the addition of l-arginine or inhibition of arginase in neutrophil/T-cell co-cultures restored T-cell proliferation in G-CSF-treated healthy donors (55) and cancer patients, respectively (49).

### Circulating Tumor Cell-Escorting Neutrophils

An emerging tumor-promoting function of circulating neutrophils has recently been unveiled. Neutrophils were shown to entrap circulating tumor cells (CTCs) at metastatic sites to facilitate their extravasation thus contributing to metastasis (56–60). Recent data showed that mouse neutrophils interacted with CTCs to promote their proliferation within the bloodstream and subsequently foster metastasis (61). In breast cancer patient blood, a high level of CTC-neutrophil clusters was associated with a higher risk of developing metastases (61).

Taken together, in addition to the NLR, there is a strong rationale for routinely monitoring CTC-neutrophil clusters with the aim of evaluating their prognostic impact and predictive value in cancer patients.

## Phenotypic and Functional Heterogeneity of Tumor-Associated Neutrophils (TANs)

Neutrophils are able to infiltrate tumor tissue and are termed tumor-associated neutrophils (TANs). In mice, TANs express CD11b^+^ Ly6C^int^ Ly6G^hi^, whereas in humans, they are identified as CD11b^+^ CD14^−^ CD66b^+^ CD15^hi^ cells (50).

### Identification and Quantification of TANs in Cancer Patients

The clinical relevance of evaluating pro- and anti-tumor functions of TANs is highly pertinent in cancer patients, since TAN infiltration was reported to predict either poor (62–66) or good prognosis (67–69). Conditions that differ between good and poor prognostic TANs will be discussed below. Although the methods of analysis of survival across studies were similar, identification of quantification methods of TANs infiltrating human tumors varied greatly. Hematoxylin & Eosin (H&E) staining remains a good approach to quantify TAN infiltration based on the unique segmented-nucleus morphology of neutrophils. Scanned-tumor slides stained with H&E are accessible from TCGA public database and have already been used for TAN quantification (70). However, this approach may under-estimate the potential infiltration of immature neutrophils since the banded-nucleus morphology of immature neutrophils is less distinguishable from other immune cells.

TAN infiltration can be also quantified through immunostaining, using antibodies against markers of neutrophils, such as CD66b and CD15. Neutrophils and eosinophils share numerous markers as they are closely related ontologically speaking, and very few studies took this cofounding effect into account. Evidence showed that CD66b is expressed at the same level between eosinophils and neutrophils in peripheral blood of patients with rheumatoid arthritis (71). For CD15 (Sialyl-Lewis X), although expressed on both eosinophils and neutrophils, data showed that it is 10–100 times higher on neutrophils than eosinophils (72). CD15 would therefore be more reliable to distinguish neutrophils from eosinophils, although the search for differentially expressed markers between these two cell types is strongly needed. Some studies also used myeloperoxidase (MPO), neutrophil elastase, CXCR2, or CD33 to identify TANs in human tumors. However, such markers are not specific to TANs and are shared by other immune cells (45, 73–76) or even tumor cells, such as CXCR2.

Bioinformatics approaches inferring the fractions of tumor-infiltrating immune cells from bulk tumor RNAseq data have recently emerged (77). The most classical approach for quantifying neutrophil infiltrate relies on single marker genes specific to neutrophils, such as CSF3R. Different groups have used this approach to classify tumors based on CSF3R expression (78, 79). Other approaches using multiple gene signatures have come to light. Although signatures slightly differ from one another study, algorithms processing these signatures vary greatly. For instance, single sample Gene Set Enrichment Analysis (ssGSEA) was recently used for the stratification of lung cancer patients based on the gene signature of a newly identified pro-tumor neutrophil population. CIBERSORT was used for quantifying neutrophil infiltrate in human tumor based on a signature composed of genes highly expressed in blood neutrophils compared to other blood leukocytes (80, 81).

### Pro-Tumor TANs

In both murine and human diseases, TANs are mostly described for their ability to promote tumor progression through different mechanisms, such as tumor cell proliferation. Mouse Gr1^+^ myeloid cells secrete interleukin-1 receptor antagonist (IL-1RA), which were shown to antagonize the anti-tumor effects of cellular senescence in a murine model of PTEN^−/−^ prostate cancer (82). Neutrophil elastase (NE), a protease secreted by neutrophils, accesses the endosomal compartment of tumor cells. There, it degrades the insulin receptor substrate 1 (IRS-1) which increases the interaction between PI3K and PDGFR, thereby promoting their proliferation in a LSL-K-ras model of murine lung adenocarcinoma and in human lung adenocarcinoma cell lines (83). Interestingly, the authors identified in human lung adenocarcinoma an inverse correlation between NE and IRS-1. Neutrophil-derived leukotrienes were also reported to selectively expand the subset of cancer cells that retained high tumorigenic potential (84). Accumulating evidence supports an important role for neutrophil extracellular traps (NETs), composed of DNA, that are associated with proteins such as NE and foster cancer progression. Mechanistically, neutrophils secrete HMGB1 during NETosis, thereby activating the TLR9 signaling pathways in cancer cells to promote their adhesion, proliferation, migration, and invasion (59). A more recent study reported that NET-derived DNA could act as a scaffold for the neutrophil DNA-associated NE and MMP9 proteins during laminin-111 (matrix protein) remodeling which activated downstream integrin α3β1 signaling in disseminated, dormant cancer cells converting them into aggressive lung metastatic cancer cells (85). In addition to their role in tumor cell proliferation, NET-derived molecules such as Cathepsin G (CG) and NE were reported to promote invasion and migration of breast cancer cells (86). Indeed, NETs were observed by intravital imaging *in vivo* in the murine 4T1-derived lung metastasis model. This finding was transferable to humans as they identified the deposition of NETs in triple-negative breast tumors. Recent evidence also implicates neutrophils in the induction of the epithelial to mesenchymal transition (EMT) by sustaining the expression of the EMT transcription factor Snail in cancer cells (87) or via the secretion of the tissue inhibitor of matrix metalloproteinase (TIMP-1) (88), which thereby facilitates metastatic progression. Evidence also suggest that neutrophils could favor angiogenesis and support tumor growth as neutrophil depletion was associated with a decreased number of developed vessels and a lower tumor weight (89). Pro-tumor TANs supporting angiogenesis, were reported to express high level of the proangiogenic factors VEGF and MMP9 in a mouse model of melanoma (89) and in liver tumorigenesis in zebrafish (90). Other groups support the fact that VEGF is highly expressed by pro-tumor TANs and may thus actively promote angiogenesis in mice (91, 92). Evidence in humans showed that fMLF-activated neutrophils induced sprouting of capillary-like structures via VEGF in an *in vitro* angiogenesis assay (93). Another group showed that human neutrophils could promote angiogenesis through NETs (94). In human gastric cancer, tumor cells were showed to make neutrophils produce MMP9, which significantly promoted angiogenic tube formation (95). Further studies in humans will be required to support the idea that proangiogenic neutrophils could promote tumor progression. In contrast, new evidence showed that TANs could foster cancer progression by altering angiogenesis instead, increasing hypoxia which in turn stabilizes the Snail EMT transcription factor, but also contributes to inhibit anti-tumor adaptive immunity (87). Moreover, evidence showed that neutrophils could impair angiogenesis by secreting antiangiogenic factors such as the isoform of VEGF-A, namely, VEGF-A_165b_ (96, 97).

TANs can also promote tumor progression by interacting with immune cells. TANs were mainly described for their ability to dampen T-cell-mediated anti-tumor immunity. In mice, depending on the tumor model, different TAN-derived soluble factors were reported to suppress proliferation and IFNγ production of intra-tumor CD8^+^ T-cells, such as ROS (91) or nitric oxide (NO) produced by inducible nitric oxide synthase (iNOS) (98). The soluble enzyme arginase 1 derived from tumor-infiltrating myeloid cells was also suggested to mediate T-cell suppression (99). A recent single cell transcriptomic study across human and mouse lung tumor-infiltrating immune cells showed that arginase 1 was mostly expressed by TANs and tumor-associated macrophages (TAMs) (100, 101). Unlike human blood neutrophils, evidence in mice showed that TANs do not secrete arginase 1 but rather seem to retain it in the cytoplasm to deplete L-arginine intracellularly (99, 102). Other TAN-soluble factors were reported to induce T-cell apoptosis via the secretion of tumor necrosis factor alpha (TNFα) and NO (103). Lately, Fas-Ligand (Fas-L) was shown to be expressed at the cell surface of TANs, leading to T-cell apoptosis in tumors resistant to T-cell based immunotherapies (104).

### Anti-tumor TANs

Though largely believed to be pro-tumor, accumulating evidence suggests that TANs also play a role in anti-tumor immunity. Indeed, TANs appear to induce direct tumor cell apoptosis by secreting cytotoxic molecules such as ROS (91, 105). Neutrophil-derived extracellular DNA (NETs) were also reported to induce tumor cell death (106). Another intriguing anti-tumor property of TANs was recently identified. TANs were found to promote the detachment of tumor cells from the basement membrane at an early stage of mouse uterine carcinogenesis, a process known as tumor cell sloughing leading to tumor cell death (78). Accumulating evidence suggests that neutrophils may kill antibody-opsonized cancer cells via antibody-dependent cell cytotoxicity (ADCC), which involves Fc receptors (107–110). Recently, an original mechanism of ADCC, a process termed trogoptosis, was described (111). Indeed, neutrophils establish a synapse with cancer cells, which strongly depends on neutrophil CD11b/CD18 integrins, allowing neutrophils to ingest a fraction of the antibody-opsonized plasma membrane of cancer cells (trogocytosis). A mechanical disruption of the plasma membrane concomitantly occurs, leading to a lytic/necrotic form of cell death. In sharp contrast with the other ADCC-related cytotoxic mechanisms mostly described in NK cells, trogoptosis is independent of granule exocytosis and of the phagocyte NADPH oxidase. Lastly and more importantly, intravital imaging demonstrated that trogoptosis occurs *in vivo* in mice (111).

Another anti-tumor role for TANs in mice and humans was uncovered with their implication in the recruitment and activation of intra-tumor CD4^+^ and cytotoxic CD8^+^ T cells (91, 112). A recent study conducted in mice genetically deficient for Tollip, an innate immunity signaling adaptor molecule inhibiting the TLR signaling pathway and potentially other pathways, led to the upregulation of STAT5 and STAT1. This in turn upregulated the co-stimulatory CD80 and downregulated the immune checkpoint PD-L1, specifically in TANs (112). These molecules are both important for T-cell proliferation, IFNγ and granzyme B production. Adoptive transfer of Tollip-deficient neutrophils slowed down colitis-associated cancer progression, thus highlighting a role for Tollip in modulating TAN-mediated cancer immune surveillance (112). In human colorectal cancer, CD66b^+^ TANs stimulate proliferation and induce secretion of IFNγ from CD8^+^ T cells *in vitro* (113). Moreover, CD66b^+^ TANs frequently co-localize with CD8^+^ T-cells in tumor tissue (113). At an early stage of human lung tumors, TANs were put forward as T-cell antigen presenting cells (APCs), with a high capacity to stimulate T-cell proliferation and IFNγ production (114, 115). A recent study conducted in a murine sarcoma model showed that TANs acting in concert with macrophages, were essential for unconventional αβ T cell type 1 polarization to display anti-tumor potential *in vivo* by secreting IFNγ (79).

### TAN Subsets

With such functional heterogeneity of neutrophils, one could hypothesize that different subsets of TANs may be involved. Previous studies on blood and bone marrow neutrophils led to the discovery of new cell-surface markers to distinguish mature from immature neutrophils. Recent evidence showed that both mature and immature neutrophils can infiltrate mouse and human tumors, and were either reported to promote or prevent tumorigenesis (53, 54, 115, 116). Density could also discriminate a subset of blood neutrophils, known as LDNs, isolated by density gradient centrifugation. This method is suitable for blood samples, though its application to tumor samples may not be possible, which remains to be investigated. The search for cell-surface biomarkers of LDNs is thus needed to evaluate the relevance of LDNs in tumors. The previously identified Lox1 marker specific to a subset of LDNs was validated *in situ* in melanoma, colon, head and neck, and non-small cell lung cancer (48). But it remains to be verified if lox1 is not intracellularly expressed by all neutrophils. Characterization of TAN subsets based on cell-surface antigens remains limited to a dozen markers, making the comparison of neutrophil subsets across tissues difficult. Therefore, it remains therefore unclear whether immature blood neutrophils are identical to immature TANs. To further characterize subsets of TANs, transcriptomic profiling of neutrophils is strongly required. A recent single cell transcriptomic study performed in lung cancer patients showed distinct subsets of TANs and blood neutrophils with few overlaps between tissues (101). Whether such a difference can be attributed to differences in processing or isolation of neutrophils between tumor tissues and blood, or whether it is a true biological difference between tissues, remains to be addressed in future investigations. This study demonstrated that different subsets of TANs may co-exist in the same tumor, some being preferentially enriched, while certain TAN subsets seem to be found exclusively either in healthy or tumor tissue (101). The authors also sequenced at the single-cell level TANs from murine lung tumors (101). Unbiased comparison between mouse and human TANs showed conserved subsets allowing scientists to test the functional relevance of distinct subsets in tumor progression and response to therapies in murine models, and eventually apply finding to cancer patients. The work of Zilionis et al. unveiled TAN heterogeneity in the lung tumor context and it remains to be determined whether the same applies other tumor types. Another major ongoing issue is TAN ontogeny, and future investigations are needed to decipher whether TAN subsets correspond to transitional cell states referring to the concept of polarization or whether they are terminally-differentiated distinct cell types, and if TANs present in the tumor microenvironment are derived from circulating G-MDSCs.

### TANs in Early vs. Later-Stage Tumors

Evidence in mice supports this idea that the pro- and anti-tumor role of neutrophils may be strongly linked to tumor stage. Mice with genetic deficiency of G-CSF-R displayed an accelerated tumor initiation at early-stage in a spontaneous murine model of uterine carcinogenesis (78) and 3-methylcholanthrene (3-MCA)-induced sarcomagenesis (79), attesting an anti-tumor role at early-stage of tumorigenesis. In contrast, antibody-mediated neutrophil depletion in established tumors at late-stage, led to decreased tumor growth, supporting a pro-tumor role at late-stage (84, 98).

**Figure 1:**
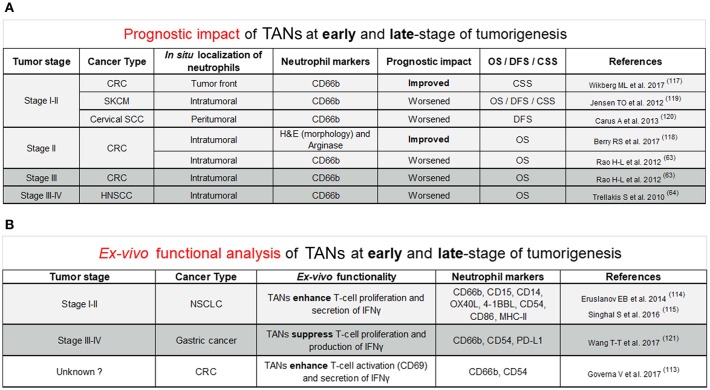
**(A)** This figure reviews studies supporting the improved or worsened prognostic impact of TANs *in situ* at early (stage I and stage II; stage II alone) and late-stage (stage III; stage III and IV) of tumorigenesis across different cancer types. Quantification neutrophils based on a particular location *in situ* was precised (intratumoral, tumor front, and peritumoral). Neutrophil markers used for identification of neutrophils *in situ* by immunostaining were either CD66b or Arginase. Others identified neutrophils *in situ* based on their morphology through Hematoxylin and Eosin (H&E) staining. Patient prognostic impact was assessed based on overall survival (OS), disease-free survival (DFS), and cancer specific survival (CSS). Light gray background refers to early-stage tumors whereas dark gray background points out late-stage tumors. Abbreviations were used for the cancer type column: CRC, Colorectal cancer; SKCM, Skin Cutaneous Melanoma; Cervical SCC, Cervical squamous cell carcinoma; HNSCC, Head and Neck squamous cell carcinoma. **(B)** This figure covers studies *ex-vivo* functional analysis supporting the anti-tumor or pro-tumor role of neutrophils in early (stage I and II) or late-stage (stage III and IV) of tumorigenesis in various cancer types. This figure includes markers expressed at protein level by anti-tumor or pro-tumor neutrophils. Light gray background refers to early-stage tumors whereas dark gray background points out late-stage tumors. White background was set for unknown tumor stage. Abbreviations were used for the cancer type column: NSCLC, Non-small-cell lung carcinoma; CRC, Colorectal cancer.

In humans, evidence for pro- and anti-tumor functions of TANs arose from retrospective studies assessing the prognostic value of tumors highly infiltrated with neutrophils (Figure 1A). The prognostic impact of TANs remains controversial as they are associated with either a better or worse outcome. Such discrepancies can be explained by the cancer type, the *in situ* location (peritumoral, intratumoral, or stromal) of neutrophils but also by differences in staining methods such as haematoxylin/eosin (HE), neutrophil elastase (NE), Arginase, MPO, CD66b, or CD15 surface markers. New evidence suggest that the tumor stage may also explain such differences (Figure 1A). In colorectal cancer (CRC) patients, the good prognostic impact of TANs is described in early-stage colorectal tumors, especially stage I–II tumors combined (117), or stage II alone (118). Only one study in CRC patients showed that a high intratumoral infiltration of TANs was associated with a worse overall survival (63). The worse prognostic impact of TANs in early-stage tumors was reported in melanoma (119) and cervical squamous cell carcinoma (120). In late-stage tumors, especially in CRC and head and neck squamous cell carcinoma, the prognostic impact of TANs is generally poor (63, 64). To our knowledge, there is no study reporting a good prognostic significance of TANs in late-stage tumors.

Other evidence supporting the link between anti- or pro-tumor function of TANs with the tumor stage came from *ex vivo* functional analysis of TANs (Figure 1B). In gastric cancer patients, TANs isolated from fresh tumors co-cultured with purified autologous peripheral blood CD3^+^ T cells displayed higher immunosuppressive function than neutrophils from healthy adjacent tissue (121). In this study, T-cell suppression is partially explained by PD-L1 expression on TANs since PD-L1 blocking antibodies reverse T-cell proliferation (121). Interestingly, the authors showed that PD-L1^+^ TANs were significantly higher in advanced (stages III–IV) vs. early-stage tumors (stage I–II), suggesting that pro-tumor PD-L1^+^ neutrophils only emerge at a later-stage of gastric tumorigenesis. Further investigations will be needed to confirm the predominance of pro-tumor TANs in late-stage gastric tumors compared to early-stage tumors. In sharp contrast, in lung cancer patients, TANs, not peripheral blood neutrophils, from early-stage tumors (stages I–II), were reported to enhance the proliferation of autologous T-cells stimulated with anti-CD3/CD28 antibodies (114, 115), suggesting an anti-tumoral function. Nevertheless, the improved prognostic impact of TANs in early-stage lung tumors remains to be assessed. In CRC, co-culture of human TANs from colorectal tumors with autologous CD8 T-cells resulted in an increased expression of the CD69 T cell activation marker and a higher release of IFNγ in culture supernatant (113). However, it remains to be determined if these CD8 T-cell-stimulatory TANs are enriched in early-stage colorectal tumors as compared to late-stage tumors. In line with the CD8 T-stimulatory anti-tumor role of neutrophils in CRC, the majority of studies on favorable outcome of TANs in early-stage tumors are in CRC (Figure 1A). Thus, it cannot be excluded that not tumor stage but rather tumor type (and possibly involvement of microbiota), is responsible for this observation, in CRC. Human undifferentiated pleomorphic sarcomas (UPS) recently emerged as another type of cancer in which dense neutrophil infiltrate was found to be associated with better prognosis, regardless of tumor stage (79). The search for cancer type-specific features, especially in CRC and UPS will be the object of future investigations to potentially identify new factors important for the stimulation of anti-tumor neutrophils.

## Origin of pro- and Anti-tumor Neutrophils

Having reviewed the heterogeneity of pro- and anti-tumor neutrophils in cancer, it is necessary to discuss their origin.

### Neutrophil Activation

The anti- or pro-tumor properties of neutrophils often result from the activation of neutrophils. Neutrophil-derived NETs were previously described to have both pro (85) and anti-tumor properties (106). *In vitro*, NETs can be generated upon acute stimulation of neutrophils with lipopolysaccharide (LPS), Phorbol 12-myristate 13-acetate (PMA), N-Formylmethionyl-leucyl-phenylalanine (fMLF formerly termed fMLP) (83, 106, 122) in both human and mice. Whereas, PMA or fMLF was sufficient to induce NETs (122). Mechanistically, LPS-stimulated platelets via TLR4 which in turn led to the activation of neutrophils thus leading to NETosis. Other platelet activators such as thrombin were found to be equally efficient in producing NETs in presence of platelets (123). The mechanism by which activated-platelets promote NETosis is still not clear and will require further investigations. Cancer cell-derived soluble molecules such as G-CSF were also suggested to induce NETs (86). The engagement of the Fc-alpha receptor (FcαRI/CD89) (124) or Fc-gamma receptor (FcγRIIIB/CD16) (125) was also reported to induce NETs. Neutrophil-derived NET formation is not restricted to *in vitro* stimulation, as it can be reproduced in mouse blood following acute systemic sepsis (58) and in mouse lung tumor after chronic nasal instillation of LPS (84). Neutrophils were also described to display T-cell suppressive functions. Both mouse and human neutrophils acutely stimulated by high doses of IFNγ, up-regulate T-cell suppressing ligands such as PD-L1 which in turn decrease T-cell proliferation (115, 126). The acute induction of endoplasmic reticulum (ER) stress upon thapsigargin (THG) stimulation in human circulating neutrophils was reported to convert these cells into T-cell suppressive G-MDSCs, notably by inhibiting T-cell proliferation (48). Interestingly, in contrast to THG treatment, fMLF and PMA-stimulated neutrophils did not block T-cell proliferation. Instead, PMA-stimulated neutrophils appear to have T-cell suppressive effects by inhibiting the production of T-cell derived IFNγ (127). Activation of the complement receptor 3 (MAC-1/CD11b) signaling pathway also seems important for the induction of T-cell suppressive neutrophils (128).

The engagement of Fc-receptors on neutrophils constitutes another way of activating neutrophils and was reported to lead to killing of antibody-opsonized cancer cells by ADCC *in vitro* (109, 111) and *in vivo* (111). Interestingly, neutrophils were shown to induced ADCC more efficiently with IgA antibodies in comparison to IgG antibodies (107, 108, 110). Using hFcαRI transgenic mice, IgA anti-EGFR antibodies were proven to mediate tumor cell killing *in vivo* (129). Recent evidences support the idea that ADCC could occur *in vivo* as revealed by intravital imaging (111). Taken together, direct acute or chronic stimulation of neutrophils seems to be sufficient to transform naïve neutrophils into pro- or anti-tumor neutrophils. Collectively, neutrophil activation may in turn lead to a variety of pro- and anti-tumor function depending on type, dose of activator, time of stimulation, and tumor model.

### Neutrophil Differentiation

By definition, differentiation implies the development of a given progenitor toward several different mature cells with distinct cell fates. Depending on environmental cues, neutrophil progenitors were reported to differentiate into different subsets of neutrophils with either pro- or anti-tumor functions (115, 130, 131).

Mounting evidence indicates that IFNγ and GM-CSF, two cytokines abundantly present in early-stage human lung tumors, are essential for the differentiation of CD11b^+^ CD15^hi^ CD66b^+^ CD16^int/low^ CD10^−^ bone marrow neutrophil progenitors into anti-tumor CD10^+^ mature MHC-II^+^ antigen presenting neutrophils, known as “APC-like hybrid neutrophils” (114, 115). Surprisingly, besides expressing APC markers, this TAN subset was shown to express markers of monocytes such as CD14. Both IFNγ and GM-CSF downregulate the expression of the Ikaros transcription factor, known to negatively regulate the development of monocytes/macrophages which may explain the acquisition of CD14 (132, 133). Interestingly, the Ikaros inhibitor, lenalidomide, synergizes with IFNγ and GM-CSF to generate APC-like hybrid neutrophils, but is unable to do so alone, suggesting that other pathways activated by IFNγ and GM-CSF are necessary for this differentiation, though these latter remain to be unraveled. Moreover, the authors showed that APC-like hybrid neutrophils could also be generated from CD11b^+^ CD15^hi^ CD66b^+^ CD16^int/low^ CD10^−^ low-density immature peripheral blood neutrophils from G-CSF-treated healthy donors (115). Since neutrophil progenitors may also circulate in the bloodstream of cancer patients (54, 55), it is therefore possible that circulating neutrophil progenitors are recruited at the tumor site to differentiate into APC-like hybrid TANs. Neutrophil progenitors used for the generation of APC-like hybrid neutrophils were isolated from bone marrow of cancer patients or from peripheral blood of G-CSF-treated healthy donors (115). It therefore remains unclear whether systemic priming of neutrophil progenitors by tumor-secreted factors, including G-CSF is a prerequisite for their differentiation into APC-like hybrid neutrophils upon exposure to IFNγ and GM-CSF at tumor site.

Recent evidence supports the concept that neutrophils need to be primed systemically to differentiate into pro-tumor TANs. A recent study in mice showed that the differentiation of bone marrow-derived hematopoietic stem and progenitor cells (HSPCs) into pro-tumor SiglecF^+^ TANs (130) required bone marrow osteoblasts. Indeed, *in vivo* cell fate mapping experiments showed that the differentiation of SiglecF^+^ TANs from bone marrow c-Kit^+^ HSPCs was abrogated in osteoblast-deficient mice, suggesting that the bone marrow is essential for the priming of pro-tumor SiglecF^+^ TANs. Extra-medullary tissues such as the spleen were also necessary for the differentiation of HSPCs into T-cell suppressive TANs (131). Splenectomy was indeed reported to blunt T-cell suppressive functions of TANs and synergistically enhanced anti-PD-L1 therapeutic efficacy. Mechanistically, the spleen of tumor-bearing mice mediates the recruitment of circulating HSPCs through CCL2/CCR2 axis, which then differentiate into T-cell suppressive G-MDSC via splenic stromal-derived GM-CSF. This is in line with previous studies showing that T-cell suppressive G-MDSCs expand in the spleen, which is most often characterized by splenomegaly (134). Collectively, although evidence in mice suggests that the systemic environment such as the bone marrow and the spleen are necessary for the differentiation of neutrophil progenitors to pro-tumor TANs, it is still unclear whether this systemic priming is sufficient for differentiation to pro-tumor TANs or whether it requires additional cues at tumor site for example, remains unknown.

In humans, systemic priming appears to be necessary for the generation of T-cell suppressive neutrophils. Indeed, a study recently showed that mature CD10^+^ neutrophils from G-CSF-treated healthy donors display T-cell suppressive properties *ex vivo*, whereas those from untreated healthy donors did not. Interestingly, CD10^+^ neutrophils from untreated healthy donors treated with G-CSF *in vitro* did not display any suppressive activities (55). This suggests that, in humans, G-CSF alone is not sufficient to induce T-cell properties on neutrophils and thus requires other probably systemic cues.

The tumor may also alter the differentiation of neutrophils for its own benefit, by interfering with their maturation toward an anti-tumor phenotype. This concept was initially demonstrated for tumor-infiltrating monocytes. Here, the maturation into anti-tumor macrophages was blocked leading to promotion of tumor growth in a murine model of hepatocellular carcinoma (HCC) (135). Accumulating evidences in humans and mice suggest that immature neutrophils infiltrate tumors, retain their immature phenotype at the tumor site and are correlated with a higher tumor burden (53, 54, 98, 116). Aside from the hypothesis that interfering with neutrophil maturation could alter their differentiation into anti-tumor neutrophils, this data suggest that at least some neutrophil progenitors may also have a naturally occurring T-cell suppressive function *per se*. Indeed, immature bone marrow neutrophils from healthy donors were reported to display spontaneous T-cell suppressive properties, although this remains to be confirmed in further studies (54). The biological characterization of immature TANs and their clinical implication should be the subject of future investigations.

## Neutrophil-Based Therapeutic Strategies

**Figure 2:**
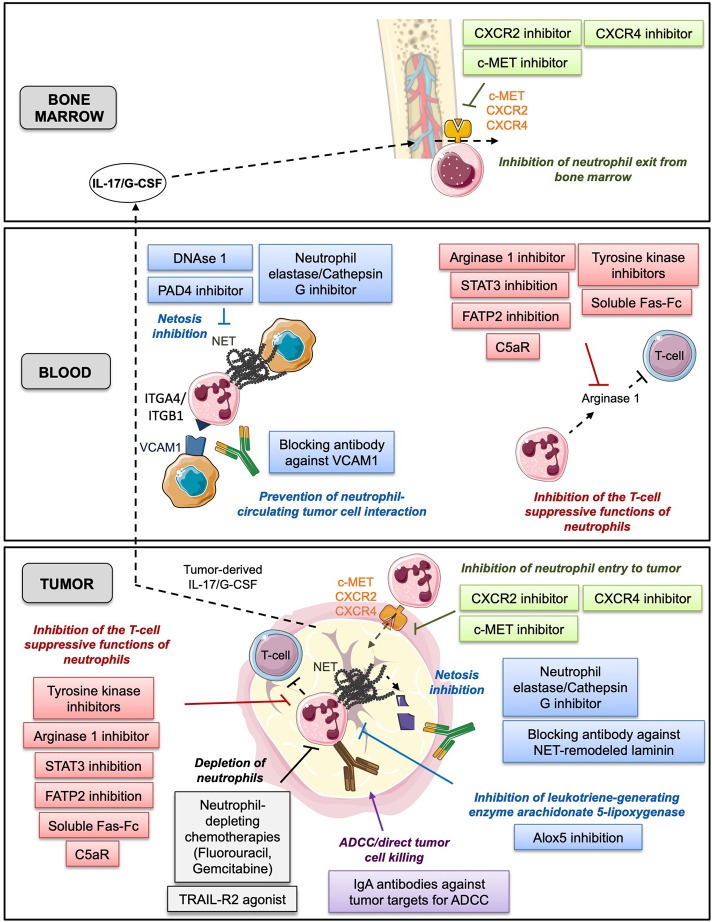
This figure summarizes the different neutrophil-based therapeutic strategies with various mechanisms of action described in bone marrow, blood and tumor. In green: prevention of neutrophil exit from bone marrow and entry to tumor tissue. In gray: depletion of neutrophils. In red: inhibition of the T-cell suppressive functions of neutrophils. In blue: prevention of neutrophil capacity to foster tumor cell proliferation and migration. In purple: promotion of the anti-tumor functions of neutrophils.

Since neutrophils display various tumor-promoting functions and are predictive of poor patient OS, one potential therapeutic strategy could be the targeting of neutrophils *in vivo*. Pre-clinical studies in the mouse have already reported therapeutic effects of neutrophil depletion using the specific neutrophil-depleting antibody, anti-Ly6G (84, 87, 98, 104). Here, we review the different therapeutic strategies that aimed at targeting neutrophils in cancer (Figure 2).

### Prevention of Neutrophil Exit From Bone Marrow and Entry to Tumor Tissue

#### G-CSF/IL-17 Axis Modulation

A number of studies in mice have suggested modulating the level of G-CSF to prevent the expansion of pro-tumor neutrophils. In mice, ablation of G-CSF with anti-G-CSF antibodies was also shown to prevent neutrophil accumulation in bloodstream (61, 98, 136), whereas overexpression of G-CSF induced the expansion of circulating neutrophils and increased metastasis (61, 136, 137). Although G-CSF is being used to stimulate the production of neutrophils in patients who suffer from chemotherapy-induced neutropenia (138), it remains unclear if endogenous G-CSF is responsible for neutrophilia observed in cancer patients. Few studies reported a higher level of serum G-CSF in patients with pancreatic cancer (139), colorectal cancer (140) and non-small cell lung cancer (141), as compared with healthy subjects. None of these studies showed an association between high serum G-CSF concentration and neutrophilia. Only few case report studies reported a link between a high endogenous serum G-CSF level and an increase of white blood cell count consisted primarily of neutrophils (142–144). Taken together, the role of endogenous serum G-CSF as a dominant driver of neutrophilia in cancer patients remains weak. Furthermore, endogenous serum G-CSF levels has not been linked to poorer prognosis to date. Noteworthy, exogenous therapeutic G-CSF does not seem either to worsen survival of cancer patients treated with chemotherapy (145, 146). Interleukin-17 (IL-17) recently emerged as an upstream regulator of G-CSF and subsequently of neutrophil production *in vivo* (98, 147, 148). In a murine model of lung metastasis, neutralization of IL-17 significantly reduced the level of G-CSF and prevented the systemic expansion of blood neutrophils. Interestingly, studies report an increase of IL-17 in the blood of patients with liver cancer (149), non-small cell lung cancer (148, 150, 151). High level of serum IL-17 was even associated with a worse prognostic for non-small cell lung cancer patients (150, 151). Nevertheless, the link between level of serum IL-17 and neutrophilia remains to be investigated in future studies.

#### CXCR2/CXCR4 Inhibition

CXCR2 is known to be important for neutrophil migration, controlling their egress from the bone marrow to the bloodstream and their recruitment to sites of inflammation (152–155). Neutrophil acquire CXCR2 to exit bone marrow (153, 154). The genetic or pharmacological inhibition of CXCR2 was reported to decrease primary lung tumor growth (156, 157) and suppress pancreatic cancer metastasis in mice (157). In both studies, specific depletion of Ly6G^+^ neutrophils recapitulated the therapeutic effect of CXCR2 inhibition. CXCR2 inhibition was followed by an increase in circulating neutrophils due to their inability to home (156, 157). Although some evidence indicates that CXCR2 inhibition could prevent recruitment of CD11b^+^ Gr1^+^ myeloid cells in PTEN^−/−^ prostate tumors (82), it remains unclear whether Ly6G^+^ neutrophil infiltration at the tumor site is impaired. Since therapeutic effects of CXCR2 inhibition may also be explained by its direct action on tumor cells (158–160), future studies are needed to determine the contribution of neutrophils to CXCR2 inhibition-related therapeutic effects. This would provide a rationale for using CXCR2 as a potent inhibitor of neutrophil recruitment.

CXCR4 was reported to act antagonistically with CXCR2 for bone marrow exit of neutrophils (153, 154, 161). Neutrophils lose CXCR4 to egress from the bone marrow to be released in the circulation (154, 161). However, CXCR4 acquisition seems important for infiltration of neutrophils into the tumor, at least in a preclinical model of colorectal cancer. Hence, blockade of CXCR4 using the FDA-approved agent Plerixafor (AMD3100) inhibited anti-VEGFR2 therapy-induced tumor infiltration of neutrophils and Ly6C^low^ monocytes (162). Increasing number of studies report the therapeutic effects of inhibiting CXCL12/CXCR4 axis by targeting cancer and stromal cells from the tumor microenvironment (163). Very few studies looked at the effects of CXCR4 blockade on tumor-infiltrating immunosuppressive myeloid cells including neutrophils and it remains the object of future investigations. Taken together CXCR4 inhibition appears to be an interesting strategy when used in combination with chemotherapy. This by preventing neutrophils from entering into the tumor while mobilizing bone marrow neutrophils which could compensate chemotherapy-induced neutropenia classically managed by G-CSF injection. Hence, several pre-clinical studies showed the additive therapeutic effects of CXCR4 inhibition with chemotherapies (164–166).

#### C-Met Inhibition

Capmatinib, an inhibitor of the tyrosine-protein kinase MET (c-MET), was first developed to treat cancer patients with alterations affecting the c-MET pathway in cancer cells due to activating mutations, overexpression, gene amplification, and translocations (167). Interestingly, capmatinib potentiated therapeutic effects of adoptive T-cell transfer and checkpoint immunotherapies in several mouse models of cancer by preventing the reactive mobilization and recruitment of T-cell suppressive neutrophils to tumors and draining lymph nodes (126). Importantly, the therapeutic effect was restricted to c-MET signaling in neutrophils as the genetic ablation of c-MET kinase activity specifically in neutrophils could reproduce the same effect. Moreover, tumor cell lines were insensitive to capmatinib *in vitro* and *in vivo*, excluding any tumor cell-intrinsic c-MET dependency in this study. Another study showed that capmatinib also prevented the recruitment of anti-tumor TANs (168) in different tumor models. Whereas, cancer cell lines knocked down for c-MET had a slower tumor growth rate in mice, the addition of capmatinib countered the therapeutic effect of c-MET knockdown, suggesting that TME-expressing c-MET were involved in the anti-tumor response. TANs appeared to express c-MET which is essential for their recruitment at the tumor site and thereby activation of their anti-tumor function. These studies therefore argue in favor of evaluating the expression of c-MET in both tumor cells and neutrophils in cancer patients who receive capmatinib treatment.

### Depletion of Neutrophils

#### TRAIL-R2 Agonist Antibody

An agonistic antibody of the TNF-Related Apoptosis-Inducing Ligand Receptor 2, known as TRAIL-R2, was proposed to induce cell death of mouse G-MDSC *in vitro* and to potentiate the effect of CTLA-4 immune checkpoint blockade *in vivo*. A recent first-in human clinical trial evaluated the clinical impact of targeting TRAIL-R2. In cancer patients with elevated levels of LDNs before treatment, the TRAIL-R2 agonist antibody selectively depleted LDNs without impacting the number of other peripheral blood myeloid and lymphoid cells, nor showing dose-limiting toxicities (169). However, the selective depletion of LDNs could not be sustained up to 28 days after the start of the treatment. Due to short-term treatment and the small number of patients in the cohort, further studies will be needed to conclude on the selective depletion of LDNs upon TRAIL-R2 agonist antibody treatment.

#### Chemotherapies

Aside from their potential cytotoxic effects on tumor cells, studies in mice revealed that some chemotherapies may also have side effects on immune cells, including neutrophils. Indeed, fluorouracil (5FU) was reported to have anti-tumor effects *in vivo* in mice and was associated with depletion of splenic and tumor G-MDSCs and monocytic MDSCs (M-MDSCs) (170). In this study, the therapeutic efficacy of 5FU regarding tumor immunity was suggested to most likely be restricted to M-MDSC-depletion since 5FU did not deplete any other immune cell type, nor induce immunogenic tumor cell death. Nevertheless, the same group reported several years later that 5FU and Gemcitabine could also drive M-MDSC-derived IL-1b secretion that induce the release of CD4 T-cell-derived IL-17, which in turn blunts the anticancer efficacy of these chemotherapeutic agents. However, gemcitabine and 5FU were shown to exert higher anti-tumor effects when tumors were established in Nlrp3^−/−^ or Casp1^−/−^ mice or wild-type mice treated with the IL-1 receptor antagonist (IL-1Ra) (171). Therefore, the depleting effect of 5FU on MDSCs, including G-MDSCs, still remains a matter of debate and requires further investigation.

Taken together, studies aiming at evaluating the neutrophil-depleting effects of already-approved therapies remain contrasted. These discrepancies are likely due to differences in doses, timing of administration, location of neutrophil sampling, time of neutrophils detection, as well as mouse and tumor models (172, 173). Future investigations should therefore take into account these parameters to identify in which settings these potential neutrophil-depleting therapies could be the most beneficial. Since neutrophils are constantly monitored during cancer treatment to prevent neutropenia and therefore avoid opportunistic infections, depletion of neutrophils must be restricted to the tumor site (TANs), or only to subsets of neutrophils with tumor-promoting function. To support the rationale that currently-used chemotherapies may offer additional therapeutic effects by targeting TANs, it will be important to determine whether a higher infiltration of TANs prior to the administration of neutrophil-depleting treatments is associated with a better survival. Interestingly, evidence in stage III colorectal cancer patients all treated with 5FU after surgical removal, showed that a high density of intratumoral TANs before treatment was associated with a longer disease-free survival (68). TAN infiltrate may therefore help identify patients who will likely benefit from 5FU chemotherapy.

### Inhibition of the T-Cell Suppressive Functions of Neutrophils

#### Arginase 1 Inhibitor

L-arginine is an important amino acid that serves as a building block for protein synthesis and as a precursor for multiple intra-cellular metabolites (174). L-arginine is known to be particularly important for T-cell proliferation and survival (175). Moreover, increased intracellular L-arginine in T-cells was shown to favor their differentiation to central memory-like T-cells with enhanced anti-tumor properties (176). L-arginine is mainly catabolized by arginase 1, which is secreted by subsets of myeloid cells, including neutrophils under specific conditions in humans (46, 55). Several reports demonstrated *in vitro* that neutrophil-derived arginase 1 suppresses T-cell proliferation (55), which was rescued by the addition of an arginase 1 inhibitor. The therapeutic effect of the arginase 1 inhibitor on tumor growth was also observed in a pre-clinical mouse model of lung cancer (99, 177). However, the tumor-promoting role of arginase 1 remains controversial as L-arginine also favors tumor cell proliferation and survival. *In vitro*, recombinant human arginase 1 induces cell cycle arrest and apoptosis of human tumor cell lines (178–182). Bioengineered PEGylated arginase 1 for which the half-life was extended through PEGylation, was shown to exert anti-tumor effects in xenograft mouse models (179, 181, 183, 184). Recent evidence suggests that the bioengineered human PEGylated arginase 1 (AEB1102) exerts additive anti-tumor effects when combined to anti-PD-1 or anti-PD-L1 in melanoma, small cell lung cancer (SCLC), and sarcoma patient-derived xenografts (183, 184). Collectively these results demonstrate that disrupting the physiological balance of L-arginine may either inhibit or promote tumor progression depending on T-cell and tumor cell susceptibility to arginine starvation. The synergistic therapeutic efficacy of either recombinant arginase 1 or arginase 1 inhibitor in combination with various chemotherapies or Pembrolizumab (anti-PD-1) is currently being tested in phase 1/2 clinical trials (NCT03371979; NCT02903914; NCT03361228; NCT03314935).

#### Tyrosine Kinase Inhibitors

In mice, splenic HSPCs were shown to contribute to the T-cell suppressive function of TANs (131) in a murine model of HCC. Low-dose of sorafenib, a c-Kit inhibitor, was associated with increased apoptosis of splenic HSPCs and reduced immunosuppressive function of TANs. Sorafenib synergizes with PD-L1 blockade. The authors showed that the therapeutic effect of sorafenib could be largely attributed to splenic HSPCs depletion as the adoptive transfer of splenic HSPCs following sorafenib treatment abrogated its effects. In contrast, sorafenib promoted expansion of tumor-promoting TANs in the HCC mouse model and therefore limit sorafenib therapeutic efficacy (185). This finding was translatable in HCC patients, since TAN infiltration was higher in patients previously treated with sorafenib prior to liver resection, compared to untreated patients.

Another tyrosine kinase inhibitor, called sunitinib, has also shown promising results in depleting pro-tumor neutrophils. In renal cell carcinoma (RCC) patients, the elevated percentage of the T-cell suppressive CD15^+^ CD14^−^ LDNs among PBMCs declined in response to sunitinib treatment. *In vitro*, sunitinib induced the death of CD15^+^ CD14^−^ LDNs in a dose-dependent manner (186). Studies in mice showed that sunitinib depleted T-cell suppressive splenic neutrophils, whereas it failed to deplete T-cell suppressive TANs (187, 188). Intra-tumor GM-CSF promotes STAT5 signaling pathway activation, which largely explains the resistance of TANs to sunitinib-induced cell death. In contrast, another group showed that sunitinib depletes tumor-associated Gr1^+^ MDSC and synergizes with a cancer vaccine to enhance antigen-specific immune responses and tumor eradication (189). However, these authors did not specify which MDSC subset (granulocytic or monocytic myeloid cells) was targeted by sunitinib.

#### C5aR Blockade

The C5a receptor (C5aR, CD88) becomes fairly well known for its role in immunosuppressive activity of myeloid cells. Lung cancer cells were found to produce C5a which could bind to C5aR expressed by myeloid cells to increase their immunosuppressive functions (190). Concentration of C5a in peripheral blood of lung cancer patients was significantly higher as compared to healthy donors. A recent pre-clinical study showed additional therapeutic effects of C5a pharmacologic inhibition in combination with PD-1 blockade unleashing anti-tumor CD8 T-cell response in a model of lung cancer (191). In this latter study, the C5a inhibitor named NOX-D21 is an l-aptamer that tightly binds to C5a and inhibits the interaction with its receptors. Another group recently reported in a pre-clinical model of squamous cell carcinoma, that the targeting of C5aR1 essentially in macrophages with a peptide antagonist (PMX-53) improved efficacy of paclitaxel chemotherapy and was associated with CD8 T cell response (192). A blocking antibody against C5aR (IPH5401) was recently developed and entered phase 1 clinical trial in combination with PD-L1 blockade for lung cancer patients (NSCLC) and head and neck patients (HCC) (NCT03665129). Recent evidence suggests that not only macrophages but also neutrophils express high level of C5aR (193). Moreover, the authors showed that IPH5401 selectively inhibited C5a-induced activation and migration of human blood neutrophils, suggesting that neutrophils may account as therapeutic target of C5aR blocking antibody.

#### STAT3 Inhibition

STAT3 transcription factor is well known to be important for the tumor-promoting activities of myeloid cells such as immunosuppression (194–197) or tumor angiogenesis (198). Clinical trials using small molecule inhibitors targeting STAT3 showed limited therapeutic effects and broad side effects (199). Other approach attempting to inhibit STAT3 signaling pathway by using STAT3 siRNA are currently evaluated in clinical trials (199). The mechanisms by which STAT3 inhibition dampens the immunosuppressive function of neutrophils are not clear. Recent evidences suggest that STAT3 activation leads to the expression of the fatty acid translocase CD36 that in turn increases the uptake of lipids and the oxidative metabolism, and subsequently the immunosuppressive function of myeloid cells (200, 201). Further studies will be needed to evaluate the impact of targeting STAT3 in tumor-promoting neutrophils.

#### FATP2 Inhibition

Recent evidences suggest that lipid metabolism could contribute to the pathological activation of G-MDSC (201, 202). Human TANs and peripheral blood G-MDSC also upregulate the expression of several lipid transporters such as CD36, Msr1, Ldlr, or Lox1, as compared to neutrophils from healthy donors. Addition of very low-density lipoproteins increased the T-cell suppressive function of MDSC *in vitro* (201). Genetic depletion of the fatty acid translocase CD36 in tumor-bearing mice delayed tumor growth *in vivo* (201). More recent *in vivo* evidence showed that genetic deletion of the SLC27A2 gene (also known as FATP2) encoding the very long-chain acyl-CoA synthetase, specifically in neutrophils using the S100a8-cre mice, abrogated tumor growth in different tumor models (202). FATP2^−/−^ TANs had decreased T-cell suppressive activity as compared to WT TANs (202). The authors further showed that the selective FATP2 inhibitor lipofermata delayed tumor growth in different preclinical mouse models and synergized with either anti-CTLA4 or anti-CSF1R antibody. Human TANs were found to have a higher intracellular lipid content as compared to blood neutrophils from matched cancer patients (202). Future investigations on human samples will be needed to evaluate the clinical relevance of targeting FATP2 in cancer patients.

#### Soluble Fas-Fc

A pre-clinical mouse study recently reported that neutrophils upregulate Fas-L which in turn triggers apoptosis of tumor-infiltrating lymphocytes, thereby causing resistance to immunotherapies based on checkpoint blockade, cancer vaccines or adoptive T-cell therapy (104). In this study, soluble Fas-Fc neutralized Fas-L *in vivo*, rendering tumors sensitive to T-cell-based immunotherapies. Future studies are required to determine whether high infiltration of Fas-L positive TANs at baseline is associated with unresponsiveness to immunotherapies.

### Prevention of Neutrophil Capacity to Foster Tumor Cell Proliferation and Migration

#### Inhibition of Netosis

A preclinical study showed that NETs promote the development and progression of liver metastases after surgical stress. Patients undergoing curative liver resection for metastatic colorectal cancer, that were characterized by an increased postoperative NET formation, had a >4-fold reduction in disease-free survival (59). Accelerated development and progression of metastatic disease was demonstrated in a murine model that recapitulated NET formation after surgical stress. Local treatment with DNAse 1 or inhibition of the enzyme peptidylarginine deaminase (PAD4), which is essential for NET formation, abolished tumor progression after surgery (59). There are current limitations for the use of the PAD4 inhibitor to prevent NET *in vivo* due to its short-half-life (86). Similar to the PAD4 inhibitor, the short half-life of NE and cathepsin G inhibitors prevents their use *in vivo* for cancer treatment (86). Recently, NET-associated proteases, NE and matrix metalloproteinase 9, were shown to sequentially cleave laminin (85). This proteolytically remodeled laminin induced proliferation of dormant cancer cells by activating integrin α3β1 signaling, and antibodies against NET-remodeled laminin 111 prevented the awakening of dormant cancer cells (85). Engineering of monoclonal antibodies against human NET-remodeled laminin would help determine if such mechanisms occur in humans and whether they are correlated with patient prognosis.

#### Arachidonate 5-Lipoxygenase (Alox5) Inhibition

Evidences showed that neutrophil-derived leukotriene support breast cancer metastasis by selectively expanding cancer cells that retain high metastatic potential (84). Pharmacologic inhibition of arachidonate 5-lipoxyenase (Alox5) by zileuton (203) could inhibit the pro-metastatic activity of neutrophils. The authors showed that both leukotriene receptor Leukotriene B4 receptor 2 (BLT2) and Cysteinyl leukotriene receptor 2 (CysLT2) were expressed in human breast tumor and matched lymph node metastases. Future investigation will be needed to determine the therapeutic potential of such targets.

#### Blockade of Neutrophil-Circulating Tumor Cell Interaction

Accumulating evidence indicates that neutrophils interact with circulating tumor cells to promote their proliferation and favor their extravasation to metastatic sites (58, 61, 204). Either NETs or VCAM1 adhesion molecules seem to be important in the interaction between neutrophils and circulating tumor cells. DNAse 1 treatment or VCAM1-deficient tumor cells prevented such interaction and impaired metastasis.

### Promotion of the Anti-tumor Functions of Neutrophils

#### Antigen-Dependent Cellular Cytotoxicity (ADCC)

Mounting evidence strongly supports the concept of targeting Fc-Receptors on neutrophils in anti-tumor immunotherapy. Although they express IgA-Fc-Receptor (CD89) and IgG-Fc-Receptors (CD16; CD32; CD64), evidence showed that neutrophils are more effective at mediating ADCC through IgA antibodies than IgG antibodies *in vitro* (107, 108, 110). Numerous pre-clinical studies conducted in CD89 transgenic mice confirmed its superior ability to induce neutrophil-dependent tumor cell killing for different tumor-associated antigens, such as HER2/neu (on breast carcinoma), EpCAM (colon carcinoma), EGFR (epithelial carcinoma and renal cell carcinoma), HLA class II (B-cell lymphoma), CD30 (T- and B-cell lymphoma), and CD20 (B-cell lymphoma) (205). Due to its shorter half-life as compared to other serum isotypes, the engineering of IgA antibodies could be a promising therapeutic option to increase effectiveness of currently given monoclonal antibody-based immunotherapies.

## Conclusion and Perspectives

Although immunotherapies held great promise for oncology, only a small percentage of cancer patients showed benefits from these treatments. The search for new therapeutic targets is thus critically needed. Current immunotherapies mostly rely on the adaptive immune system, involving adaptive immune checkpoints such as CTLA-4, PD-1, and PD-L1. Recent pre-clinical studies suggest that innate immunity could offer new therapeutic opportunities. Inhibition of the innate immune checkpoints Tyro3, Axl, and Mertk tyrosine kinase receptors, mostly expressed by TAMs, displayed additional therapeutic effects when combined to the anti-PD-1 monoclonal antibody in a murine model of triple-negative breast cancer (206). Accumulating evidence suggests that neutrophils may be ideal targets for future therapeutic strategies. Several groups reported that the selective depletion of neutrophils could delay tumor growth. Ongoing pre-clinical investigations aim at evaluating the potential synergistic effect of neutrophil depletion with currently approved immunotherapies. In cancer patients, accumulating evidence from retrospective studies supports a rationale for eliminating neutrophils. Indeed, several approved cancer drugs were shown to deplete neutrophils and were associated with a better survival. Nonetheless, we cannot rule-out off-target therapeutic effects of these drugs. Moreover, blood neutrophil count is routinely used as a read-out of cancer treatment-related toxicities, since neutrophils are important in the immune response upon bacterial infection. Further studies are therefore needed to identify pro-tumor neutrophil-associated targets non-overlapping with antibacterial neutrophils, to enhance therapeutic efficacy of immunotherapies while minimizing side effects.

While the elimination of neutrophils seems to provide therapeutic effects in established tumor models, emerging evidence favors an anti-tumor role for neutrophils in early-stage tumors in mice (78, 114, 115). Evidence from retrospective studies and *ex vivo* functional analyses also support the concept of an anti-tumor role for neutrophils in early-stage human tumors. Although challenging, access to early-stage tumors will be original and fundamental to gain new insights into potentially novel yet undiscovered phenotypes and functions of TANs. With such diverse roles, the ideal targeting of neutrophils in oncology would be to promote the enrichment of anti-tumor neutrophils while depleting pro-tumor ones without altering antibacterial neutrophils.

The current dogma in cancer immunotherapy mainly relies on restoring anti-tumor T-cell response. However, a high tumor infiltration by anti-tumor lymphocytes is not sufficient to eradicate all tumor clones during the course of metastasis as for example in colorectal cancer patients, shedding light on tumor-intrinsic and tumor-extrinsic mechanisms of escape that remain to be discovered (207). Resistance to CD8 T cell-induced tumor cell death by ferroptosis can be one possible tumor cell-intrinsic mechanism of escape (194). Neutrophils can kill tumor cells in a non-apoptotic mechanism by a process called trogoptosis, distinct from ferroptosis by its lytic/necrotic form (208) of cell death that may release DAMP and tumor (neo) antigens from the killed cancer cells (111). Neutrophils could therefore improve cytotoxic T cell response to eliminate resistant tumor clones.

Beyond their role at tumor site, neutrophils seem to promote tumor progression by acting systemically as exemplified by the ability of blood neutrophils to escort circulating tumor cells within the bloodstream and facilitate their extravasation to metastatic site (56–61). Moreover, the T-cell suppressive activity of circulating neutrophils is restricted to *in vitro* studies. It remains to be known if such neutrophil-mediated T-cell suppression occurs in blood *in vivo*. Growing evidences also highlight the importance of distant microenvironment away from tumor bed such as the bone marrow and the spleen to educate pro-tumor TANs (130, 131). Future studies should therefore consider the importance of the different microenvironments outside of the tumor bed, regarding the origin and function of neutrophils in cancer, for the benefits of patients.

## Author Contributions

PL, MS, MP, and JM wrote the original draft of manuscript. PL performed original figures and overall the supervision of writing. LK, CC, NB-V, and M-CM revised and edited the manuscript.

### Conflict of Interest Statement

The authors declare that the research was conducted in the absence of any commercial or financial relationships that could be construed as a potential conflict of interest.
